# Impact of surgical correction of tetralogy of fallot on short-term right and left ventricular function as determined by 2-dimensional speckle tracking echocardiography

**DOI:** 10.1097/MD.0000000000004426

**Published:** 2016-08-07

**Authors:** Yuman Li, Xinfang Wang, Qing Lv, Jing Wang, YaLi Yang, Lin He, Li Yuan, Li Zhang, Mingxing Xie

**Affiliations:** Department of Ultrasound, Union Hospital, Tongji Medical College, Huazhong University of Science and Technology, Wuhan, China.

**Keywords:** speckle tracking echocardiography, tetralogy of Fallot, ventricular function

## Abstract

Right ventricular (RV) and left ventricular (LV) dysfunction is an important determinant of poor clinical status in repaired patients with tetralogy of Fallot (TOF). The purpose of our study is to assess the impact of surgical repair on short-term RV and LV function by 2-dimensional speckle tracking echocardiography (STE).

Sixty-seven patients (median age 12 months) with TOF before and 6 months after repair and 35 healthy subjects were studied. The patients were divided into the younger (age at surgery ≤12 months) and older (age at surgery >12 months) subgroups. RV and LV global longitudinal systolic strain and strain rate (SR), and LV global circumferential and radial systolic strain and SR were measured by STE. After repair, RV longitudinal strain and SR increased in the younger patients, whereas RV longitudinal SR was decreased in the older patients. LV deformation parameters were unchanged in all patients. In the multivariate analysis, patients with better RV and LV deformation parameters preoperatively were identified to have better RV and LV strain and SR postoperatively (*P* < 0.05 for all). The surgical approach of the pulmonary valve ring was predictive of RV and LV systolic function postoperatively (*P* < 0.05 for all).

After TOF repair, short-term RV function improvement is identified in the younger but not in the older patients, whereas LV function is unchanged in all patients. The preoperative RV and LV deformational indices are the determinant of postoperative biventricular function improvement. STE appears to be a valuable tool for assessment of biventricular function after congenital heart disease surgery.

## 1 Introduction

Tetralogy of Fallot (TOF) is the most common cyanotic congenital heart defect. Survival of patients with TOF has steadily increased since the introduction of intracardiac surgery, with early mortality <2%.^[1]^ Most patients with TOF have an uneventful postoperative course; however, a small number of patients experience a troublesome postoperative recovery. Geva et al^[2]^ demonstrated that right ventricular (RV) and left ventricular (LV) systolic dysfunction was an important determinant of poor clinical status of survivors of TOF repair. Therefore, the investigation of the short-term effects of TOF repair on RV and LV function is of vital importance because the early identification of individuals at greatest risk and timely intervention will most likely improve clinical outcomes. Previous studies focus predominantly on the long-term RV and LV function after TOF repair.^[2,3]^ Furthermore, very limited data are available on the short-term impact of TOF repair on RV function evaluated by tissue Doppler imaging (TDI).^[4]^ It is well known that TDI has an intrinsic limitation of angle-dependence, which could less accurately assess myocardial performance.

Recently, 2-dimensional speckle tracking echocardiography (STE) is an emerging method to evaluate myocardial performance. The STE-derived strain and strain rate (SR) have been demonstrated as more sensitive markers for detecting early changes in myocardial performance than conventional echocardiographic parameters.^[5]^ Moreover, STE could be widely used in the evaluation of RV and LV function in patients with a variety of cardiovascular diseases,^[6–8]^ including congenital heart diseases.^[9]^ To the best of our knowledge, no studies have been performed to determine the impact of surgical correction of TOF on short-term RV and LV function as assessed by STE.

Therefore, the objective of the present study was to investigate the short-term effects of TOF repair on RV and LV function using STE and to explore the possible factor that might predict postoperative RV and LV myocardial function improvement.

## 2 Methods

### 2.1 Study population

From June 2008 to November 2014, 67consecutive patients with TOF were scheduled for intracardiac repair in our hospital (40 males, 27 females; median 1 year, range 3 months–18 years) and were enrolled in our study. Four patients were in New York Heart Association (NYHA) functional class I, 53 patients in NYHA functional class II, and 10 patients in NYHA functional class III. Inclusion criteria were included: concomitant small atrial septal defects or patent foramen ovale. The patients with ≥1 following criteria were excluded: pulmonary atresia, atrioventricular septal defect, residual intracardiac shunt, RV—pulmonary arterial connection by conduit. The patients were divided into younger (age at surgery ≤12 months, 41cases, median 10 months, range 3 months–1 year) and older (age at surgery >12 months, 26 cases, median 3 years, range 16 months–18 years) subgroups.

The control group consisted of 35 age- and sex-matched normal subjects (median 1 year, range 2 months–18 years; 24 males) who had no evidence of cardiopulmonary diseases through physical check-up, electrocardiogram, chest X-ray, and echocardiography. All subjects were determined to be in sinus rhythm. The study was approved by the local research ethics committee at Union hospital, Tongji medical college, Huazhong University of Science and Technology, China. The subjects or their parents in our study gave informed consent.

### 2.2 Echocardiography

All echocardiographic parameters were obtained before and 6 months after repair. Echocardiography was performed using a commercially available ultrasound transducer and equipment (M3S and M7S probes, Vivid 7; GE Medical Systems, Horten, Norway). Right ventricular end-diastolic diameter (RVEDD) was determined from the apical 4-chamber view. The peak gradient across the right ventricular outflow tract (RVOT) was measured using continuous-wave Doppler from the RVOT view. For conventional RV function assessment, the tricuspid annular peak systolic velocity (Sm), early-diastolic velocity (Em) and late-diastolic velocity (Am) assessed by TDI, and the tricuspid annular peak systolic excursion (TAPSE) obtained from M-mode echocardiography were measured from the apical 4-chamber view at the RV free wall level.^[10]^ RV end-diastolic area (EDA) and end-systolic area (ESA) were also measured from the apical 4-chamber view to calculate RV fractional area change (RVFAC).^[10]^ The degree of pulmonary regurgitation (PR) was graded as mild (no retrograde diastolic flow in pulmonary trunk with detectable regurgitant jet in the RVOT), moderate (retrograde diastolic flow in main pulmonary artery), or severe (additional retrograde diastolic flow in branch pulmonary arteries).^[11]^ Left ventricular end-diastolic volume (EDV), end-systolic volume (ESV), and ejection fraction (EF) were measured by biplane Simpson method.

**Figure 1:**
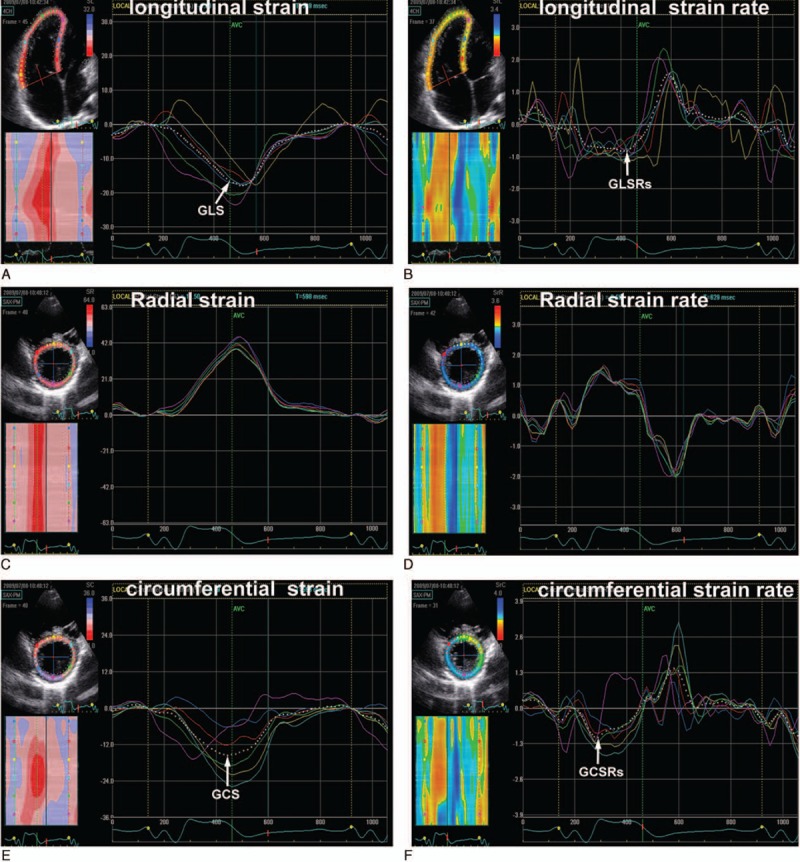
Right ventricular longitudinal (A, B), left ventricular radial (C, D) and circumferential (E, F) strain and strain rate curves in patients with tetralogy of Fallot. The white dotted curves represent the global longitudinal and circumferential strain and strain rate. GCS = global circumferential systolic strain, GCSRs = global circumferential systolic strain rate, GLS = global longitudinal systolic strain, GLSRs = global longitudinal systolic strain rate.

Two-dimensional grayscale images of subjects at a frame rate of 60 to 90 frames/s were obtained from the apical 4-chamber view and parasternal LV short-axis view at mid-ventricular level. RV and LV longitudinal strain and SR were measured from the apical 4-chamber view; LV radial strain and SR, and circumferential strain and SR were measured from the LV mid-ventricular short-axis images. All images were stored for off-line analysis (EchoPAC, version BT06; GE-Vingmed, Norway). For speckle tracking analysis, the RV and LV endocardial borders were manually traced in the end-systolic frame at the point in the cardiac cycle in which the endocardial border was the clearest. The region of interest in each image was automatically generated. The position of the region of interest and its width was adjusted manually when the speckle tracking appeared to be poor. The software automatically tracked and accepted segments of good tracking quality and rejected poorly tracked segments. When all segments of the RV or LV were accepted, the RV and LV longitudinal strain and SR curves and the LV radial and circumferential strain and SR curves were automatically generated. RV and LV global longitudinal systolic strain (GLS) and strain rate (GLSRs), and LV global circumferential systolic strain (GCS) and strain rate (GCSRs) were automatically obtained from the strain and strain rate curves. LV global radial systolic strain (GRS) and strain rate (GRSRs) referred to the average of the LV 6 segments at the mid-ventricular short-axis images. Representative examples of RV and LV strain and SR curves in patients with TOF are shown in Figure 1. All measurements were performed 3 times, and the mean value was used for analysis.

### 2.3 Statistical analysis

Statistical analysis was performed with a SPSS software package (SPSS for Windows 11.5, SPSS Inc, Chicago, IL). Continuous data were expressed as mean value ± standard deviation or median. Comparison among the preoperative and postoperative values was made with Student paired *t* test. Correlations between biventricular global systolic function improvement postoperatively and the preoperative parameters (including biventricular dimension, biventricular systolic deformation parameters, LVEF, RV conventional systolic function parameters [Sm, RVFAC, and TAPSE] and QRS duration), the surgical approach of the pulmonary valve ring level, clinical variables (age at repair, sex, heart rate), and the degree of PR were calculated as Pearson or Spearman correlation coefficient depending on data distribution. Multivariate stepwise linear regression analysis was used to test the possible role of the above preoperative parameters, the surgical approach of the pulmonary valve ring level, clinical variables, and the degree of PR in the prediction of biventricular function improvement after surgery. Intraobserver and interobserver variability were assessed in randomly selected 15 subjects. Intraobserver variability was determined by having 1 observer remeasurement after 2 months. Interobserver variability was determined by a second observer who was blinded to the clinical and the STE findings. Interobserver and intraobserver reproducibility were evaluated by means of intraclass correlation coefficient (ICC). For all analyses, a value of *P* < 0.05 was considered as statistically significant.

## 3 Results

### 3.1 Clinical and conventional echocardiographic characteristics

Clinical and conventional echocardiographic parameters before and 6 months after repair are listed in Table 1. All surgeries were performed using cardiopulmonary bypass and deep hypothermia. For the surgical therapy at the pulmonary valve ring level, 38 patients had been operated on using transannular patches, and nontransannular patches were used in 29 patients. Three patients were lost to follow-up. One patient died on the fifth day after repair. Three patients were excluded owing to suboptimal image quality. Thus, the final TOF group consisted of 60 patients (39 younger and 21 older patients). After repair, 51 patients were in NYHA functional class I and 9 patients in NYHA functional class II. We identified 28 patients with mild, 8 with moderate, and 24 with severe PR. Mild tricuspid regurgitation was also present in 19% patients early after surgery. After repair, the gradient across the RVOT was significantly decreased in all patients. RVEDD, RVEDA, and RVFAC had an increased tendency but without statistical significance in the younger and older patients. The QRS duration was longer in all patients. The younger and older patients showed decreased TAPSE, Sm, and Am. The increased LVEDV and LVESV were identified in the younger and older patients. In contrast, LVEF did not change postoperatively.

**Table 1:**
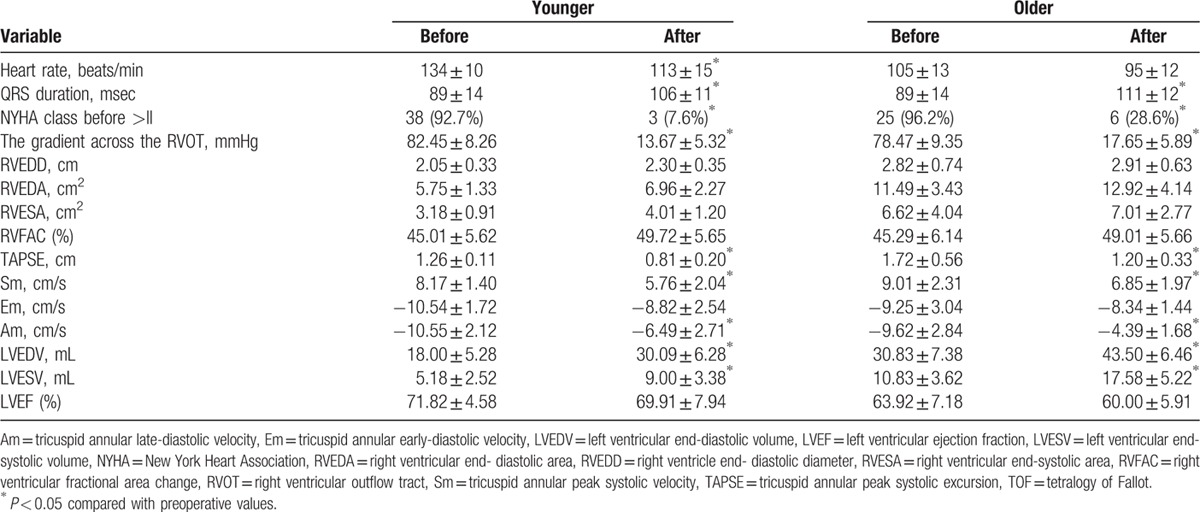
Clinical and conventional echocardiographic parameters before and 6 months after TOF repair.

### 3.2 RV and LV systolic function before and after repair

The changes in RV and LV global strain and SR before and after repair in the younger and older patients are presented in Table 2. RV GLS and GLSRs decreased in the younger and older patients preoperatively compared to those of healthy controls. Compared with controls, there was a decrease in the LV global radial strain in the younger and older patients preoperatively. After repair, RV global strain and SR increased in the younger patients, whereas RV global SR was decreased in the older patients. LV global longitudinal, circumferential, and radial strain and SR were unchanged in the younger and older patients. RV and LV deformation indices after repair were lower in patients with transannular patches than in those with nontransannular patches.

**Table 2:**
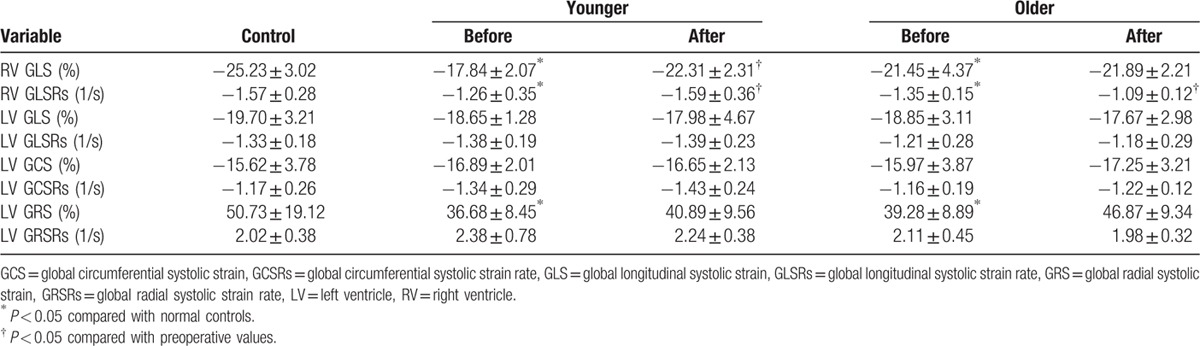
Right ventricular and left ventricular systolic strain and strain rate before and after repair.

### 3.3 Right–left ventricular interactions before and after repair

Before repair, RV GLS was associated with LVEDV (*r* = 0.435, *P* = 0.038). We also identified the correlation between LV deformation parameters and RVEDA and RVESA in our study (*P* < 0.05 for all).

After repair, a correlation between RV GLS and LV GLSRs and LV GCS was found in our study (*r*_1_ = 0.657, *P*_1_ = 0.002; *r*_2_ = –0.489, *P*_2_ = 0.021). Similarly, RV GLSRs was related to LV GLSRs, GCSRs, and GRSRs (*r*_1_ = 0.821, *P*_1_ < 0.001; *r*_2_ = 0.624, *P*_2_ = 0.004; *r*_3_ = 0.613, *P*_3_ = 0.005). LV GLSRs was correlated with RVEDA and RVESA (*r*_1_ = –0.598, *P*_1_ = 0.006; *r*_2_ = –0.645, *P*_2_ = 0.004). Likewise, LV GRSRs was associated with RVEDA and RVESA (*r*_1_ = –0.486, *P*_1_ = 0.027; *r*_2_ = –0.523, *P*_2_ = 0.021). The degree of PR was related to LV GRSRs (*r* = –0.478, *P* = 0.029).

### 3.4 Association between the preoperative parameters and change in RV and LV function after repair

**Figure 2:**
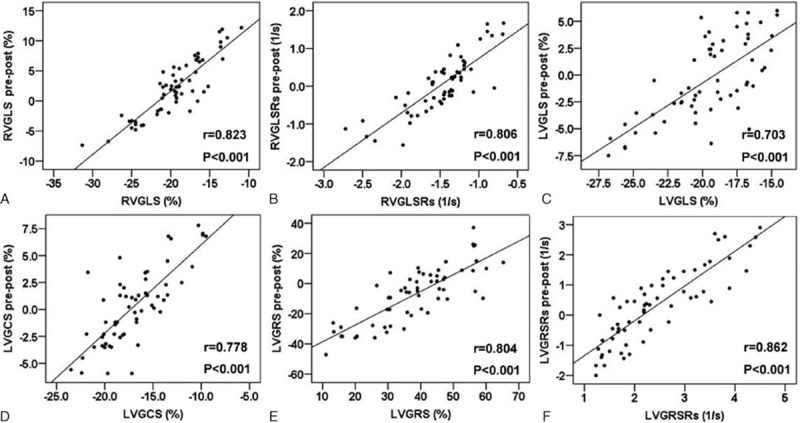
The association between the preoperative RVGLS and RVGLSRs and the change in RVGLS (A) and RVGLSRs (B) after repair; the correlation between the preoperative LV GLS and GCS and the change in LV GLS (C) and GCS (D) after surgery; the relationship between the LV GRS and GRSRs before repair and the change in LV GRS (E) and GRSRs (F) after surgery. GCS = global circumferential systolic strain, GLS = global longitudinal systolic strain, GLSRs = global longitudinal systolic strain rate, GRS = global radial systolic strain, GRSRs = global radial systolic strain rate.

The age at repair was related to LV GLSRs and GCSRs after repair in our study (*r*_1_ = –0.682, *P*_1_ < 0.001; *r*_2_ = –0.632, *P*_2_ = 0.001). Similarly, a significant correlation between postoperative RV GLSRs and the age at repair was found in our study (*r* = –0.623, *P* = 0.001). The relationship between the preoperative RV and LV deformation parameters and change in RV and LV function after surgery is depicted in Figure 2. Better RV GLS and GLSRs preoperatively correlated with better RV GLS and GLSRs after surgery, respectively (*r*_1_ = 0.823, *P*_1_ < 0.001; *r*_2_ = 0.806, *P*_2_ < 0.001). Likewise, better LV GLS and GCS preoperatively were related to better LV GLS and GCS after repair, respectively (*r*_1_ = 0.703, *P*_1_ < 0.001; *r*_2_ = 0.778, *P*_2_ < 0.001). Better LV radial strain and SR preoperatively were associated with better LV radial strain and SR after repair, respectively (*r*_1_ = 0.804, *P*_1_ < 0.001; *r*_2_ = 0.862, *P*_2_ < 0.001). However, the patients who had the most improvement were those with worse function preoperatively, as shown in Figure 2.

In the multivariate analysis, the better LV circumferential and radial strain, and RV longitudinal strain and SR preoperatively could predict better LV and RV strain and SR postoperatively, respectively (β_1_ = 0.712, *P*_1_ = 0.002; β_2_ = 0.878, *P*_2_ = 0.0001; β_3_ = 1.231, *P*_3_ < 0.001; β_4_ = 1.856, *P*_4_ < 0.001). The surgical approach of the pulmonary valve ring level (transannular or nontransannular patches) was the predictor of RV and LV longitudinal SR, and LV circumferential strain postoperatively (β_1_ = –0.618, *P*_1_ = 0.001; β_2_ = –0.438, *P*_2_ = 0.009; β_3_ = –0.648, *P*_3_ = 0.003).

### 3.5 Reproducibility of the STE measurements

The interobserver ICC was 0.95 for RV GLS, 0.94 for RV GLSRs, 0.93for LV GRS, 0.92 for LV GRSRs, 0.94 for LV GCS, and 0.91 for LV GCSRs. The intraobserver ICC was 0.96, 0.95, 0.94, 0.93, 0.95, and 0.93 for the RV GLS, RV GLSRs, LV GRS, LV GRSRs, LV GCS, and LV GCSRs, respectively.

## 4 Discussion

To the best of our knowledge, this is the first study evaluating the short-term effects of surgical correction of TOF on RV and LV performance using 2-dimensional STE. Our study demonstrates that differences exist in LV and RV adaptation after repair. The short-term RV function improvement is identified only in the younger but not in the older patients, whereas LV function is unchanged in all patients. We also identify that preoperative LV and RV deformational indices and the surgical approach of the pulmonary valve ring level are predictive of LV and RV function improvement after repair. The patients with the most improvement in deformation after repair are those with worse RV function preoperatively. STE may sensitively detect the differences in LV and RV recovery after TOF repair, indicating that it may be a valuable tool in the investigation and follow-up of patients with congenital heart disease after surgery.

### 4.1 The effect of TOF repair on short-term RV function

In the present study, we found that RV deformation parameters increased only in the younger patients after repair, whereas RV deformation indices decreased in the older patients after surgery. We speculate that postoperative RV function recovery in the older patients may be later than that in the younger patients. In our previous study,^[12]^ we identified that preoperative myocardial remodeling adversely affected the postoperative RV function as assessed by STE in the older but not in the younger patients, which further support our results in the present study. Farah et al^[4]^ showed that RV myocardial isovolumic acceleration as evaluated by TDI remained significantly lower 3 month after surgery compared with preoperative values. These findings are in agreement with our results in the older patients because their patients had high mean and median ages at surgery (39.6 and 23 months). Other studies also observed that older age at the time of surgical repair was associated with the postoperative deterioration of RV myocardial function.^[9,13]^ Our findings show the earlier surgical repair (patients younger than 12 months) favors postoperative RV function recovery, which was advocated by cardiac surgeons in most centers.

Our study shows that RV function improvement after TOF repair differs depending on preoperative disease course, which is discordant with the previous reports showing the effect of pulmonary valve replacement (PVR) on RV function. Knirsch et al^[14]^ found decreased RV strain 1 month after RVR, which, although increased at 6 months, still remained lower than the preoperative values and the values from the controls. In contrast, Kutty et al^[15]^ observed that RV deformation indices using velocity vector imaging shows mild improvement after PVR, but is not normalized. Sabate Rotes et al^[16]^ showed that RV systolic and diastolic deformational parameters were unchanged after PVR. Therefore, we conclude that the responses of pressure versus volume load relief on RV function are different.

### 4.2 The effect of TOF repair on short-term LV function

Our study also provided the opportunity to study the short-term effects of TOF repair on LV function. Similar to the findings of Moiduddin et al,^[17]^ who did not observe a change in LV strain and SR after transcatheter percutaneous pulmonary valve implantation, we did not find short-term effects of TOF repair on LV function. Our results are also consistent with those of Sabate Rotes et al,^[16]^ who did not find a change in LV strain and SR after PVR. Therefore, we could conclude the similar responses of RV pressure versus volume load relief on LV function. The absence of significant improvement in LV deformation parameters after repair may be because of the fact that strain and SR are remodeling-dependent parameters where one would not expect significant short-term changes after repair.^[18]^

### 4.3 Ventricular–ventricular interactions

That the RV influences LV function should not be surprising given the anatomic context of shared myofibres encircling both ventricles, a common ventricular septum and a single pericardial cavity. Evidence for hemodynamic impact of the RV on the LV, and vice versa, has been demonstrated in previous study.^[19]^ Unfavorable ventricular–ventricular interactions were identified in our study. An association between RV and LV systolic deformation parameters after repair was found, similar to previous studies.^[20]^ We also identified a close correlation between chamber size and contralateral ventricular function before and after repair, different from previous studies.^[16]^ A correlation between the degree of PR and LV radial strain rate was also found in our study. This finding is consistent with the study of Fernandes et al,^[21]^ which demonstrated that higher PR volume was associated with decreased LV radial strain.

### 4.4 The determinant of RV and LV performance after repair

In the present study, we observed that postoperative RV and LV systolic performance was correlated with age at repair. These findings are similar to those of previous studies.^[9,13,22]^ Our results indicate that early repair of TOF is advised to minimize the effect of long-standing hypoxia and pressure overload. Geva et al^[2]^ demonstrated that an older age at TOF repair was the strongest independent factors associated with poor clinical status. Therefore, cardiac surgeon suggests early repair of TOF, which has an advantage on postoperative biventricular recovery and improving long-term prognosis.

In the multivariate analysis, patients with better RV and LV deformation parameters preoperatively were identified to have better RV and LV systolic function after repair, suggesting that STE based on myocardial deformation may help refine the decision-making process in these patients. Our results are consistent with the findings of Sabate Rotes et al,^[16]^ who found that preoperative LV and RV deformation was associated with LV and RV function after PVR. In addition, we also showed that the surgical approach of the pulmonary valve ring level was predictive of postoperative RV and LV function improvement. RV and LV deformation indices were lower in patients with transannular patches than in those with nontransannular patches, similar to previous studies.^[9,23]^ Our results indicate that trying to avoid transannular patch during the operation is very important to improve RV and LV function postoperatively.

### 4.5 Limitations

Our study has some limitations. First, cardiac magnetic resonance imaging is the criterion standard of RV volume and function; the absence of cardiac magnetic resonance assessment is one shortcoming of our study. Second, speckle tracking analysis software that is originally designed for left ventricle was applied to the RV analysis in the present study. Therefore, deformational changes of the RVOT, wherein more or less surgery was performed, could not be assessed using 2-dimensional STE.

A third limitation of this study is that it is a single-center study. Surgical techniques may differ from center to center. Different surgical techniques may impact postoperative RV or LV function. A fourth limitation is the lack of 3-dimensional echocardiography, which provides more appropriate information about RV and LV volume and function postoperatively compared with 2-dimensional echocardiography.

Finally, our study included a relatively small number of patients, and future studies with larger samples are warranted to determine the effect of surgical correction on long-term biventricular function in patients with TOF.

## 5 Conclusion

Assessment of myocardial function using STE demonstrates that the postoperative remodeling process of myocardial function is different for the LV and the RV. The short-term RV function improvement postoperatively is only identified in the younger but not in the older patients, whereas LV systolic function is unchanged after surgery. Patients with better preoperative LV and RV deformation parameters have better LV and RV systolic function after repair. The surgical approach of the pulmonary valve ring level has been demonstrated to predict RV and LV function improvement postoperatively. Therefore, STE appears to be a valuable quantitative tool for follow-up evaluation of biventricular performance after congenital heart disease surgery.
